# A High Malaria Prevalence Identified by PCR among Patients with Acute Undifferentiated Fever in India

**DOI:** 10.1371/journal.pone.0158816

**Published:** 2016-07-07

**Authors:** Christel Gill Haanshuus, Sara Chandy, Anand Manoharan, Rosario Vivek, Dilip Mathai, Deepika Xena, Ashita Singh, Nina Langeland, Bjørn Blomberg, George Vasanthan, Usha Sitaram, Jonathan Appasamy, Joel Nesaraj, Anil Henry, Suvarna Patil, Gerardo Alvarez-Uria, Lois Armstrong, Kristine Mørch

**Affiliations:** 1 National Centre for Tropical Infectious Diseases, Department of Medicine, Haukeland University Hospital, Bergen, Norway; 2 Infectious Diseases Training and Research Center, Department of Medicine Unit-1 and Infectious Diseases, Christian Medical College, Vellore, India; 3 Baptist Christian Hospital, Tezpur, Assam, India; 4 Department of Clinical Science, University of Bergen, Bergen, Norway; 5 Christian Fellowship Hospital, Oddanchatram, Tamil Nadu, India; 6 Bethesda Hospital, Ambur, Tamil Nadu, India; 7 Christian Hospital, Mungeli, Chhattisgarh, India; 8 B.K.L. Walawalkar Hospital, Ratnagiri, Maharashtra, India; 9 Rural Development Trust Hospital, Anantapur, Andhra Pradesh, India; 10 Duncan Hospital, Raxaul, Bihar, India; Quensland University of Technology, AUSTRALIA

## Abstract

**Background:**

Approximately one million malaria cases were reported in India in 2015, based on microscopy. This study aims to assess the malaria prevalence among hospitalised fever patients in India identified by PCR, and to evaluate the performance of routine diagnostic methods.

**Methods:**

During June 2011-December 2012, patients admitted with acute undifferentiated fever to seven secondary level community hospitals in Assam (Tezpur), Bihar (Raxaul), Chhattisgarh (Mungeli), Maharashtra (Ratnagiri), Andhra Pradesh (Anantapur) and Tamil Nadu (Oddanchatram and Ambur) were included. The malaria prevalence was assessed by polymerase chain reaction (PCR), routine microscopy, and a rapid diagnostic test (RDT) with PCR as a reference method.

**Results:**

The malaria prevalence by PCR was 19% (268/1412) ranging from 6% (Oddanchatram, South India) to 35% (Ratnagiri, West India). Among malaria positive patients *P*. *falciparum* single infection was detected in 46%, while 38% had *P*. *vivax*, 11% mixed infections with *P*. *falciparum* and *P*. *vivax*, and 5% *P*. *malariae*. Compared to PCR, microscopy had sensitivity of 29% and specificity of 98%, while the RDT had sensitivity of 24% and specificity of 99%.

**Conclusions:**

High malaria prevalence was identified by PCR in this cohort. Routine diagnostic methods had low sensitivity compared to PCR. The results suggest that malaria is underdiagnosed in rural India. However, low parasitaemia controlled by immunity may constitute a proportion of PCR positive cases, which calls for awareness of the fact that other pathogens could be responsible for the febrile disease in submicroscopic malaria.

## Introduction

Malaria is one of the leading infectious causes of morbidity and death. The World Health Organization (WHO) estimated 214 million malaria cases and 438,000 malaria deaths globally in 2015 [1]. In India 1,102,205 malaria cases and 561 deaths were reported in 2015 based on microscopy and rapid diagnostic tests (RDT) [1]. Less than two malaria cases per thousand individuals per year (Annual Parasite Index (API)) is reported in most parts of India, lowest in South-, North- and West, and highest in Central-, East- and North-East [2]. However, data from malaria surveillance are uncertain since a majority of the population live in poverty in rural areas and has limited access to diagnostic services, and recent studies have shown that malaria prevalence and case fatality is underreported by surveillance systems [2, 3].

Routine malaria diagnostic methods have several limitations compared to polymerase chain reaction (PCR). Accurate microscopy requires skilled personnel and high quality technical equipment [4], and commercially available RDTs differ widely in sensitivity and specificity [5]. Both methods fail to detect low-level parasitaemia, and are inferior in optimal species identification [4, 5]. In principle, PCR can detect parasitaemia as low as one gene copy, and allows differentiation of all five *Plasmodium* species [6, 7]. In point of care diagnosis, PCR cannot replace the traditional diagnostic methods, as the technique is relatively costly, resource-demanding and time-consuming. However, using PCR as a reference method provides more accurate information about prevalence and species distribution [8, 9]. Furthermore, within the variety of PCR protocols, the choice of gene target influences the sensitivity of PCR. Targeting the mitochondrial genome yield higher sensitivity than the common 18S gene due to a higher number of gene copies per parasite [10, 11].

The primary objective of this study was to assess the proportion of malaria infections among patients with acute undifferentiated fever, presenting to secondary level community hospitals at multiple sites across India, using a highly sensitive and specific PCR targeting mitochondrial DNA as a reference method [11]. The secondary objective was to evaluate the sensitivity, specificity and species-specificity of routine microscopy and RDT compared to PCR.

## Materials and Methods

### Study design and population

The present work was part of a multi-centre, observational, cross sectional study, investigating the proportion of acute undifferentiated fever attributable to different infections. From June 2011 to December 2012, 1564 patients admitted to seven hospitals in rural or semi-urban areas in six different states of India, were enrolled prospectively and consecutively. Inclusion criteria was inpatients aged ≥5 years with temperature ≥38°C for 2–14 days prior to admission, with no localized causes of fever. Patients received health care according to routines at the participating hospitals, and no additional interventions were performed as part of the study.

### Study sites

**Fig 1:**
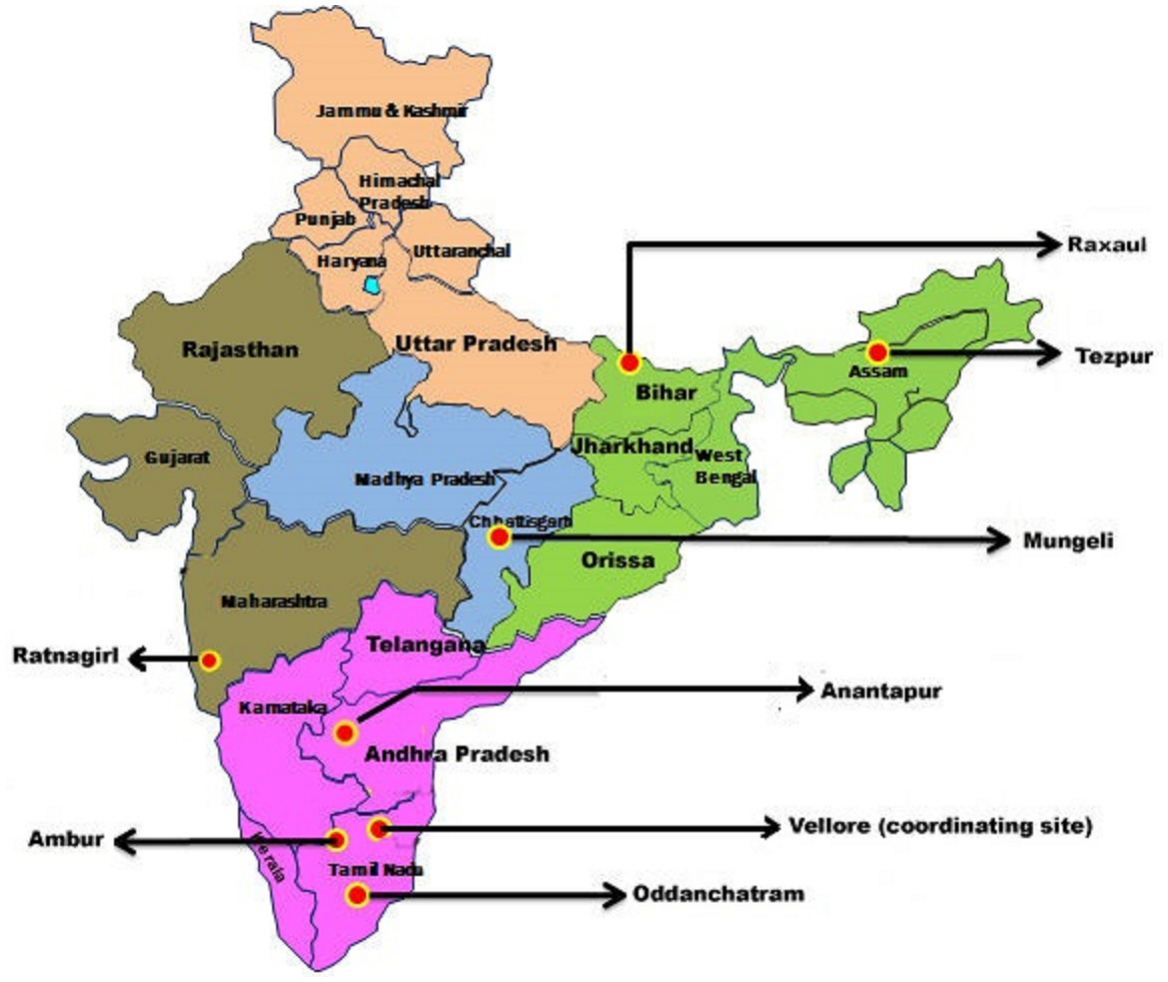
Location of seven community hospitals in six states of India participating in the study.

The study sites were located in Tezpur (Assam, North East India), Raxaul (Bihar, East India), Mungeli (Chhattisgarh, Central India), Ratnagiri (Maharashtra, West India), Anantapur (Andhra Pradesh, South India), Oddanchatram and Ambur (Tamil Nadu, South India). Fig 1 shows the locations of the study sites. These are secondary level community hospitals with 100 to 500 beds. The rainy seasons vary between the sites, as outlined in the result section presenting seasonal variations. The Benjamin M Pulimood Laboratories for Infection and Inflammation, Department of Medicine Unit 1 and Infectious Diseases, Christian Medical College, Vellore, India served as study coordinating centre and reference laboratory.

### Study procedures

Blood (0.5–1 ml and 3–5 ml from paediatric and adult patients, respectively) was drawn into EDTA tubes and stored at -20°C at the study sites before they were bulk shipped on dry ice to the coordinating centre, where molecular and antigen testing for malaria was performed. Test analyses were performed blinded from other test results. Peripheral blood smears were prepared and examined for malaria parasites at the study sites as part of the routine microscopy work-up according to the hospitals procedures. Technicians were trained and involved in routine smear examination at the respective sites, and were retrained at the reference laboratory during the start of the study. Quality control slides were sent to the sites and reported back to the reference laboratory in a satisfactory manner.

DNA for PCR analysis was extracted from 200 μl whole blood using QIAamp DNA Blood Mini Kit (Qiagen, Hilden, Germany) according to the manufacturer’s instructions, and stored at -20°C prior to application. All samples were screened for presence of *Plasmodium* DNA by a genus-specific pan-malaria PCR assay targeting the mitochondrial genome. The assay was done as previously described [11], but with a primer concentration of 1μM. In case of discordant results between PCR, RDT or routine microscopy, the samples were retested by the genus-specific PCR from the extraction step as a quality control. A malaria infection was confirmed if two or all of the three repeat PCRs were positive.

All genus-specific PCR positive samples were further analysed by a species-specific PCR targeting the 18S of *P*. *falciparum*, *P*. *vivax* and *P*. *malaria*. A modified version of the original protocol by Padley et al. [12] was used as described previously [11]. Samples negative by the species-specific PCR, were repeated by the genus-specific PCR and the PCR products were thereby sequenced for species identification as described previously [11]. The sequencing could potentially identify all five species including *P*. *ovale* and *P*. *knowlesi*.

Amplifications were done on AB Applied Biosystem veriti 96 well Thermal cycler (Applied Biosystems, Carlsbad, CA, USA), and products detected by electrophoresis on a 2% SeaKem agarose gel (Lonza, Rocland, ME, USA) stained with ethidium bromide.

The EDTA-blood samples, stored at -20°C, were also tested with the RDT ParaHIT-Total Ver. 1.0 Device 55IC204-10 (Span Diagnostics Ltd, Surat, India) at the reference laboratory following manufacturer’s instructions. It detects *P*. *falciparum* specific Histidine-Rich-Protein II and aldolase antigen of pan-malaria species (*P*. *falciparum*, *P*. *malariae*, *P*. *vivax*, *and P*. *ovale*). The test card has two regions, ‘Pf’ and ‘Pan’. A red band in the ‘Pf’ region alone indicates that the sample is reactive for *P*. *falciparum* (usually in case of low parasitaemia). Red bands in both ‘Pf’ and ‘Pan’ region indicate either single infection by *P*. *falciparum* or a mixed infection of *P*. *falciparum* with *P*. *vivax*, *P*. *ovale* or *P*. *malariae*. Appearance of a red band in the ‘Pan’ region alone indicates that the sample is reactive for infection by a malaria species other than *P*. *falciparum*. The RDT kits were quality checked using known positive and negative controls.

### Statistical analysis

Confidence intervals for tests’ sensitivities, specificities, positive and negative predictive values were calculated using the cii command in Stata 14 (StataCorp, College Station, TX, USA), and presented as exact 95% confidence intervals.

### Ethical approval

The study was approved by the Institutional Research Board at Christian Medical College, Vellore, Tamil Nadu (No. 7242 dated 11^th^ of August 2010) and by the Regional Ethics Committee of Norway (2010/2271-5). Written, informed consent was obtained from the patients.

## Results

**Fig 2:**
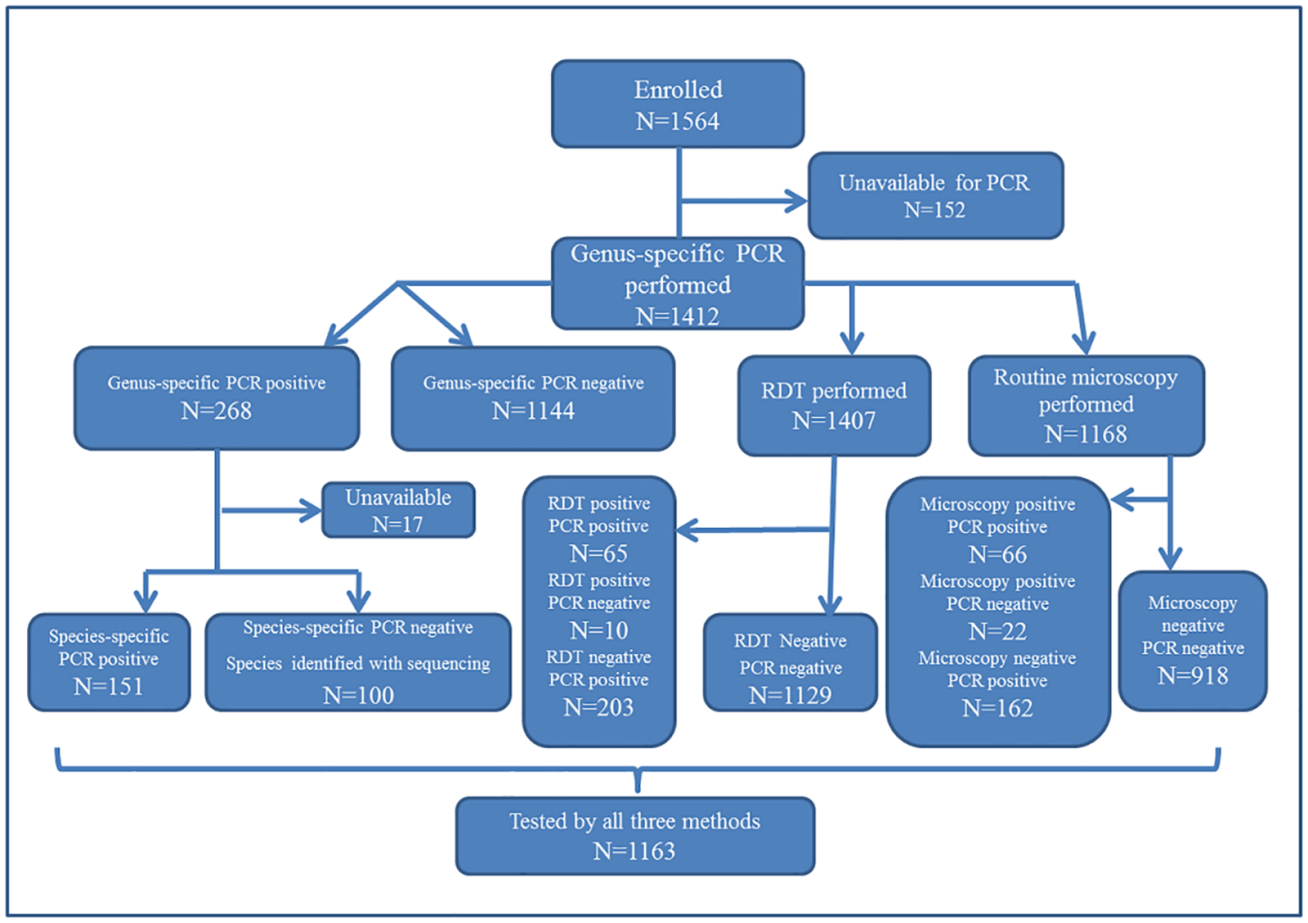
Flowchart showing investigations performed based on results and samples available.

Among patients enrolled in the acute fever study (N = 1564), samples from 1412 patients were available for malaria PCR testing, and these patients were included in the analyses. Fig 2 shows a flowchart of the investigations performed based on results and samples available. Among the 1412 patients, 815 (58%) were men, and 584 (41%) women. Mean (median) age was 34 (32) years, and 177 (13%) patients were ≤ 14 years old.

### Prevalence of malaria by PCR, all species

Genus-specific PCR identified a malaria prevalence of 19% (268/1412) as shown in Table 1. The prevalence varied from 6% (19/318) in Oddanchatram in South India to 35% (85/245) in Ratnagiri in West India.

**Table 1:** Malaria prevalence by PCR among patients admitted with acute undifferentiated fever to seven community hospitals across India, (N = 1412).

Sites	N*	Malaria PCR positives	
		N	(%)
**Total**	**1412**	268	19
**Ratnagiri** (West India)	**245**	85	35
**Raxaul** (East India)	**106**	30	28
**Mungeli** (Central India)	**52**	13	25
**Anantapur** (South India)	**124**	28	23
**Tezpur** (North-East India)	**293**	49	17
**Ambur** (South India)	**274**	44	16
**Oddanchatram** (South India)	**318**	19	6

*Total number of samples examined with malaria genus-specific PCR.

**Fig 3:**
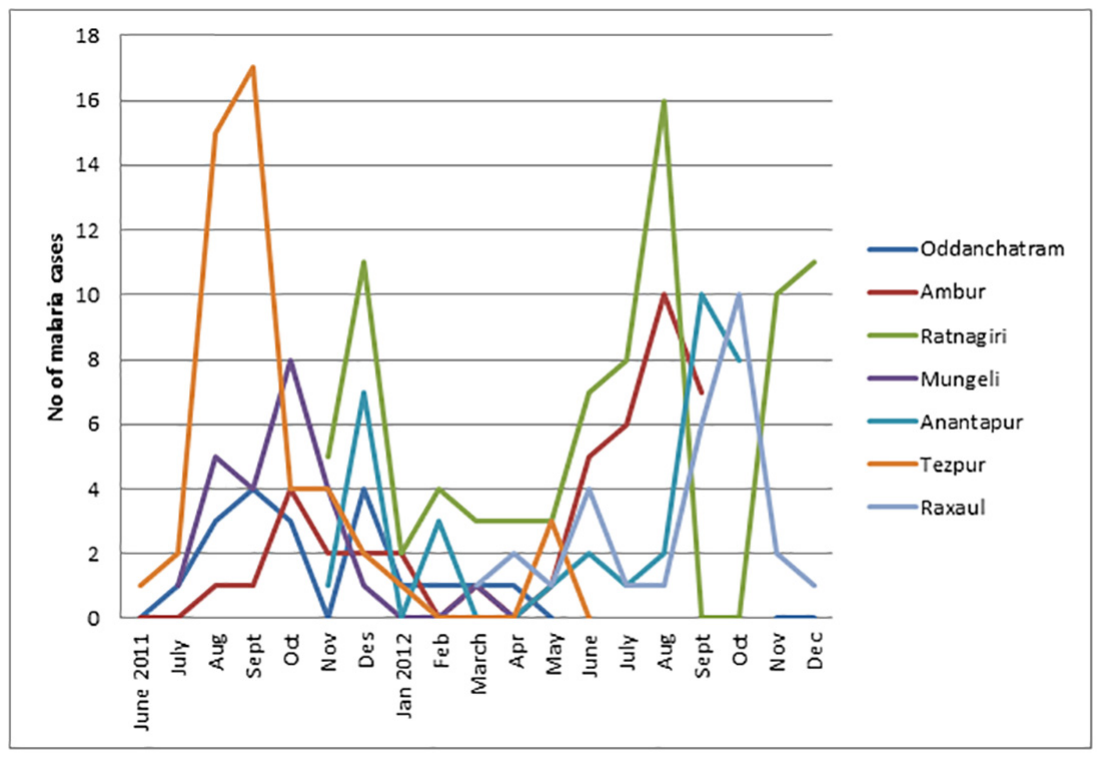
Seasonal variation among malaria PCR positive cases. The sites have the following rainy seasons: Oddanchatram; June to December, with peak monsoon from October to December. Ambur; June to December, with a peak monsoon from October to December. Ratnagiri; June to November. Mungeli; June to September, or early October. Anantapur; Dry climate, but rainy season from May to October, with its peak in September. Tezpur; April to September, with peak monsoon in July and August. Raxaul; July to September, with peak monsoon in August.

Seasonal variations in malaria prevalence is shown in Fig 3. Increased number of PCR positive cases were seen during or short after the rainy seasons at most of the sites.

### Species distribution by PCR

Species determination by species-specific PCR or sequencing was performed on samples from 251 patients (Fig 2). Overall, *P*. *falciparum* single infection was detected in 46%, while 38% had *P*. *vivax* single infections, and 11% had mixed infections with *P*. *falciparum* and *P*. *vivax*. Species distribution at the sites is presented in Table 2. *P*. *falciparum* dominated in Mungeli (Central India), Raxaul (East India) and Anantapur (South India). In Ambur (South India), *P*. *vivax* accounted for the majority. In Ratnagiri (West India), Oddanchatram (South India) and Tezpur (North-East India) the difference in *P*. *vivax* and *P*. *falciparum* distribution was less prominent. Mixed infections with *P*. *falciparum* and *P*. *vivax* were detected in a high number of patients in Anantapur, Ambur, and Tezpur. *P*. *malariae* was found at all sites except Mungeli and Raxaul. *P*. *ovale* was not detected in any of the patients.

**Table 2:** Malaria species distribution by PCR, (N = 251).

Sites	N*	P.f		P.v		P.f+P.v		P.m		P.f+P.m		P.v+P.m	
		N	(%)	N	(%)	N	(%)	N	(%)	N	(%)	N	(%)
**Total**	**251**	116	(46)	96	(38)	27	(11)	9	(4)	2	(1)	1	(0.4)
**Mungeli**	**13**	11	(85)	1	(8)	1	(8)	0		0		0	
**Raxaul**	**19**	15	(79)	4	(21)	0		0		0		0	
**Anantapur**	**27**	14	(52)	7	(26)	5	(19)	1	(4)	0		0	
**Tezpur**	**44**	20	(46)	15	(34)	6	(14)	3	(7)	0		0	
**Ratnagiri**	**85**	37	(44)	39	(46)	7	(8)	0		1	(1)	1	(1)
**Oddanchatram**	**19**	8	(42)	6	(32)	1	(5)	3	(16)	1	(5)	0	
**Ambur**	**44**	11	(25)	24	(55)	7	(16)	2	(5)	0		0	

**Abbreviations**: P.f, *Plasmodium falciparum*; P.v, *Plasmodium vivax*; P.m, *Plasmodium malariae*.

*Total number of malaria genus-specific PCR positive samples available for species determination by either species-specific PCR or sequencing.

### Performance of routine microscopy and RDT compared to PCR

Among the 1412 samples analysed by PCR, 1168 samples were analysed with routine microscopy and 1407 with RDT (Fig 2).

The total malaria prevalence detected by microscopy and confirmed positive by PCR was 6% (66/1168); at Ratnagiri 12% (29/267), Raxaul 2% (2/100), Mungeli 28% (2/5), Anantapur 8% (9/113), Tezpur 5% (14/276), Ambur 6% (7/117) and Oddanchatram 1% (3/318). Among these malaria patients, 92% (61/66, missing values 1/66) were adults (≥ 14 years old), and among PCR positive patients with negative microscopy, 85% (137/162, missing values 10/162) were adults. Compared to PCR the sensitivity of routine microscopy was 29% (Table 3). Among false positive blood slides, 18/22 was from one site, indicating high specificity of routine microscopy in six of the seven hospitals.

**Table 3:** Performance of routine microscopy and RDT compared to PCR.

	Routine microscopy	RDT
	(N = 1168)	(N = 1407)
	Percentage, 95% CI, n/total	Percentage, 95% CI, n/total
**Sensitivity**	29% (23%-35%), 66/228	24% (19%-30%), 65/268
**Specificity**	98% (96%-99%), 918/940	99% (98%-100%), 1129/1139
**Positive predictive value**	75% (65%-84%), 66/88	87% (77%-93%), 65/75
**Negative predictive value**	85% (83%-87%), 918/1080	85% (83%-87%), 1129/1332

Compared to PCR the sensitivity of the RDT was as low as 24%, while only 1% (10/1407) was false positive (Table 3). The total prevalence of malaria detected by RDT confirmed positive by PCR was 5% (65/1407); at Ratnagiri 7% (17/242), Raxaul 3% (3/106), Mungeli 12% (6/52), Anantapur 7% (9/124), Tezpur 5% (16/292), Ambur 4% (11/274) and Oddanchatram 1% (3/317).

The species concordance between PCR and routine microscopy is presented in Table 4. Microscopy was more likely to correctly identify *P*. *vivax* than *P*. *falciparum*. None of the *P*. *malariae* infections were detected by microscopy.

**Table 4:** Malaria species concordance between PCR and routine microscopy results, (N = 1168).

			Routine microscopy					
			P.f	P.v	P.f+P.v	P.o	Positive^1^	Neg
			(N = 26)	(N = 50)	(N = 5)	(N = 1)	(N = 6)	(N = 1080)
**PCR**	**P.f**	**(N = 100)**	14	8	1	1	1	75
	**P.v**	**(N = 81)**	3	21	3	0	2	52
	**P.f+P.v**	**(N = 20)**	1	4	1	0	1	13
	**P.m**	**(N = 8)**	0	0	0	0	0	8
	**P.f+P.m**	**(N = 2)**	0	0	0	0	0	2
	**P.v+P.m**	**(N = 1)**	0	1	0	0	0	0
	**Positive**^1^	**(N = 16)**	2	1	0	0	1	12
	**Negative**	**(N = 940)**	6	15	0	0	1	918

**Abbreviations:** P.f, *Plasmodium falciparum*; P.v, *Plasmodium vivax*; P.o, *Plasmodium ovale*; P.m, *Plasmodium malariae*.

Data shown for all patients examined by both PCR and microscopy.

^1^Species identification not available.

Table 5 presents species identification by RDT compared to PCR. Only one mixed infection was detected and RDT misidentified seven *P*. *falciparum* malaria infections as non-*falciparum*. None of the *P*. *malariae* single infections were detected by RDT.

**Table 5:** Malaria species concordance between PCR and RDT results, (N = 1407).

			RDT				
			P.f	Pan	P.f+Pan	Positive^2^	Neg
			(N = 9)	(N = 37)	(N = 27)	(N = 2)	(N = 1332)
**PCR**	**P.f**	**(N = 116)**	6	3	19	0	88
	**P.v**	**(N = 96)**	0	23	2	0	71
	**P.f+P.v**	**(N = 27)**	0	4	1	0	22
	**P.m**	**(N = 9)**	0	0	0	0	9
	**P.f+P.m**	**(N = 2)**	0	0	0	0	2
	**P.v+P.m**	**(N = 1)**	0	1	0	0	0
	**Positive**^1^	**(N = 17)**	1	3	2	0	11
	**Negative**	**(N = 1144)**	2	3	3	2	1129

**Abbreviations:** P.f, *Plasmodium falciparum*; P.v, *Plasmodium vivax*; P.m, *Plasmodium malariae*.

Data shown for all patients examined with both PCR and RDT.

^1^Species identification not available.

^2^Species identification not recorded.

S1 Table shows a comparison of the concordance among the three methods (N = 1163). Negative RDT among patients positive both by routine microscopy and PCR, was found in 38% (25/66), while negative microscopy among those positive both by RDT and PCR was found in 31% (18/59). Only 5% (2/43) were negative by PCR among those positive both by microscopy and RDT.

## Discussion

In this study, among hospitalized patients with acute undifferentiated fever in six states of India, we report a malaria prevalence as high as 19% using a sensitive PCR [11], compared to a prevalence of 6% identified by routine microscopy confirmed by positive PCR.

There are to our knowledge no previous hospital based malaria prevalence studies using PCR from the areas in the present study, and only a limited number of microscopy based reports. Two studies from Maharashtra (West India) among hospitalised fever patients reported a malaria prevalence of 10% (44/448) and 12% (144/1197) [13, 14], while the present study found a prevalence of 35% in Ratnagiri, Maharashtra. From Assam (North-East India) one multicentre study reported a malaria prevalence of 30% (97/324) among hospitalised fever patients [15], which is higher than the prevalence of 17% found in Tezpur, Assam. No hospital based studies are available from Chhattisgarh (Central India) where we found a prevalence of 25% in Mungeli, however, high malaria prevalence is reported in community based surveys from the neighbouring states Madhya Pradesh (Central India) and Orissa (East India) [16–18]. High prevalence, 16% and 23%, was found in the southern sites Ambur (Tamil Nadu) and Anantapur (Andhra Pradesh) in line with a study from a tertiary care hospital in Tamil Nadu reporting a malaria prevalence of 17% among fever patients [19].

During the last 30 years there has been an increasing incidence of *P*. *falciparum* compared to *P*. *vivax* in India, which has been attributed to chloroquine resistance in *P*. *falciparum* [20, 21]. Our findings supported this trend; PCR identified 46% single *P*. *falciparum* versus 38% single *P*. *vivax* infections. Predominance of *P*. *falciparum* is reported in West-, Central-, East- and North East India, and predominance of *P*. *vivax* in North- and South India [1], partly in line with the present study, where Ambur (Tamil Nadu) had the highest proportion of *P*. *vivax* (55%), and predominance of *P*. *falciparum* was found in the sites in Andhra Pradesh, Bihar, Chhattisgarh and Assam. However, also in Oddanchatram in Tamil Nadu the proportion of *P*. *falciparum* was higher than of *P*. *vivax*. Predominance of *P*. *falciparum* is expected among hospitalized malaria patients who are more prone to have severe disease, and species distribution in this study is not representative for malaria in the community population. However, the finding of *P*. *falciparum* predominance in the majority of the sites underlines the importance of considering *P*. *falciparum* aetiology in febrile patients in all parts of India. Furthermore, a high proportion of double infections (12%) were detected as supported by previous PCR studies in India [8, 9, 22].

The sensitivity detecting malaria infections by routine microscopy in the present study was only 29%, supported by several other field studies reporting low sensitivity of routine microscopy compared to PCR [23–25]. One study using PCR among fever patients in Orissa in India, reported a malaria prevalence of 81% by PCR compared to 43% by microscopy [8]. In a review of studies comparing PCR and microscopy, PCR detected on average twice as many malaria infections [26]. The low sensitivity by microscopy can be due to suboptimal staining, poor quality and inadequately maintained microscopes, and microscopists who are insufficiently trained or fatigued by high workload. However, even under optimal conditions microscopy-based diagnosis does not achieve the low detection limits that PCR-based methods yield. The PCR applied in the present study has a sensitivity of at least 0.5 parasites/μl [11], and an experienced person in a reference laboratory would not be expected to detect parasitaemia lower than 50 parasites/μl by microscopy [4]. Furthermore, the low species-specificity of microscopy compared to PCR in the present study is supported by similar findings in previous PCR studies from India [8, 9, 22].

In the present study RDT had a sensitivity as low as 24% compared to PCR, and >50% of the PCR positives which were negative by RDT were *P*. *falciparum*. The RDT ParaHIT-Total Device (Span Diagnostics) was chosen as it was widely used in routine diagnostics, easily available, reasonably priced, and could be stored at room temperature (25°C). In 2014 a study from India reported 70% sensitivity of this RDT compared to microscopy in detecting *P*. *falciparum* [27]. The sensitivity of the *P*. *falciparum* specific RDT ParaHIT *f* (Span Diagnostics) compared to microscopy ranged from 11% and 30%, respectively, in two Tanzanian studies [28, 29], to 85% in an Indian study [30]. According to the WHO’s evaluation of RDTs, the ParaHIT-Total Device detect 200 *P*. *falciparum* parasites/μl with a detection score of 85% [5], supporting that malaria with low parasitaemia probably constitute a proportion of the cases detected by PCR in this cohort.

The low sensitivity of microscopy and RDT compared to PCR may have two potential causes with impact on case management: True low sensitivity of routine diagnostics or asymptomatic parasitaemia in patients with other infections. Challenges regarding routine diagnostics in resource poor settings has been described above. A challenge using PCR in clinical diagnosis is that the method potentially also detects low parasitaemia in semi-immune individuals and not the true cause of fever. The pathogen actually causing the febrile disease may thereby be overlooked. In the present study, submicroscopic malaria was detected by PCR in 71% (162/228), and the level of asymptomatic malaria, and another infection responsible for the febrile disease, among these is unknown. Submicroscopic and asymptomatic malaria is not restricted to high endemic regions; increased prevalence has been reported from areas of low transmission intensity [31–34], and varying prevalence of asymptomatic malaria from different parts of India has been reported. Based on microscopy, two studies in tribal populations in Eastern India reported a prevalence of asymptomatic malaria of 8% and 25% respectively [35, 36], while two studies among pregnant women attending antenatal clinics in Jharkhand (East India) and Chhattisgarh (Central India) reported prevalence of asymptomatic parasitaemia as low as 1% (21/1985) and 0.5% (12/2457) respectively [37, 38]. Submicroscopic malaria is reported to be more common in adults due to the effect of acquired immunity [31, 39, 40]. The proportion of malaria identified only by PCR was not higher among adults than children in the present study.

In addition to the probability of parasitaemia controlled by immunity, a proportion of the patients in this cohort potentially suffering from a nonmalarial cause of fever, could have positive malaria PCR explained by other mechanisms. PCR may remain positive for several weeks after effective malaria treatment, which is probably explained by residual asexual parasites and/or gametocytaemia [33]. The mitochondrial PCR is particularly sensitive in detecting gametocytes because this parasite stage harbour up to eight mitochondria organelles, compared to the ring stage which harbours only one organelle [41]. In order to further elucidate the clinical impact of PCR positive malaria, quantitative real-time PCR, and gametocyte specific reverse transcriptase PCR, are methods that can be performed [42, 43]. In clinical practice in malaria endemic areas, other causes of febrile disease, such as bacterial sepsis, should be ruled out also when malaria parasites are detected, especially when there is low parasitaemia.

Although there is a risk for over-diagnosing malaria as the cause of febrile disease by PCR, the fact that the method detects low density parasitaemia, undetected by microscopy and RDT, underlines the need for accessible molecular tools for better diagnosis and estimations of the true malaria burden in India. Further, in control and elimination strategies, PCR is essential for detection of submicroscopic and asymptomatic malaria which contribute to the infectious reservoir of the disease [31, 33, 39, 40].

A potential limitation regarding the distribution of malaria across the sites is that a proportion of the patients may have contracted malaria in another area than they were hospitalised. Further, occurrence of selection bias in the inclusion of patients and samples in the favour of clinically suspected malaria cannot be ruled out. Ineffective adherence to routines in a PCR laboratory can result in false positivity due to contamination. Contamination can occur in all steps of the procedure, but it is especially important to limit accumulation of amplification products in the laboratory environment [44]. Substantial effort was given to preparations and training to set the PCR contamination risk to a minimum, with focus on executing the different steps in separate rooms, strict routines for maintaining a sterile environment, correct handling of samples etc. All genus-specific PCR results with discordancy with either RDT or microscopy were re-tested from the DNA extraction step, as a quality control.

## Conclusions

This multi-centre study from secondary level community hospitals in six states in India, reports a high prevalence of malaria among patients admitted with acute undifferentiated fever, applying PCR as the reference method. Routine microscopy and RDT had low sensitivity and species-specificity compared to PCR. As PCR is often not available or feasible in routine diagnostics, RDT and microscopy remains the mainstay in work-up of fever patients. The results of this study calls for awareness of the importance of quality assurance of malaria routine diagnostics. In work up of hospitalised febrile patients it should also be taken into consideration that patients with low parasitaemia might serve as a reservoir for transmission rather than suffering from clinical malaria, and other potential causes of fever should be ruled out.

## Supporting Information

S1 Dataset Data on patients and malaria analyses. (SAV)

S1 Table The sensitivity and specificity of three diagnostic methods detecting malaria, among the patients were all methods were performed (N = 1163). (PDF)
