# Correction: A High Malaria Prevalence Identified by PCR among Patients with Acute Undifferentiated Fever in India

**DOI:** 10.1371/journal.pone.0193574

**Published:** 2018-02-23

**Authors:** Christel Gill Haanshuus, Sara Chandy, Anand Manoharan, Rosario Vivek, Dilip Mathai, Deepika Xena, Ashita Singh, Nina Langeland, Bjørn Blomberg, George Vasanthan, Usha Sitaram, Jonathan Appasamy, Joel Nesaraj, Anil Henry, Suvarna Patil, Gerardo Alvarez-Uria, Lois Armstrong, Kristine Mørch

## Notice of republication

This article was republished on January 24, 2018 to replace an incorrect version of S1 Dataset. Please download this article again to view the correct version.
