# Supplementary material for: A High Malaria Prevalence Identified by PCR among Patients with Acute Undifferentiated Fever in India
Source: PLoS One. 2016 Jul 7;11(7):e0158816. doi: 10.1371/journal.pone.0158816 (PMC4936667; doi:10.1371/journal.pone.0158816)
Supplement: S1 Table — (PDF) [file pone.0158816.s002.pdf]

S1 Table. The sensitivity and specificity of three diagnostic methods detecting malaria, among the patients were all methods were performed (N=1163).

No. of cases with the following test results:							
**RDT^+ RM^+ PCR^+**	**RDT^+ RM^- PCR^+**	**RDT^- RM^+ PCR^+**	**RDT^- RM^- PCR^+**	**RDT^+ RM^- PCR^-**	**RDT^- RM^+ PCR^-**	**RDT^+ RM^+ PCR^-**	**RDT^- RM^- PCR^-**
41 (3.5%)	18 (1.5%)	25 (2.1%)	144 (12.4%)	7 (0.6%)	20 (1.7%)	2 (0.2%)	906 (77.9%)

Abbreviations: RM, routine microscopy; RDT, rapid diagnostic test; PCR, polymerase chain reaction.
